# Case Report: Delayed hemobilia caused by hepatic artery pseudoaneurysm with arterio-biliary fistula after liver transplantation

**DOI:** 10.3389/fmed.2026.1940000

**Published:** 2026-10-13

**Authors:** Qianlang Ren, Xianmei Wang, Dejia Xiong, Yanxin Zhao, Guoqing Wang, Junyan Lv, Lanqing Ma

**Affiliations:** 1 Yunnan Institute of Digestive Disease, The First Affiliated Hospital of Kunming Medical University, Kunming, Yunnan, China; 2 Kunming Medical University, Kunming, Yunnan, China

**Keywords:** arterio-biliary fistula, case report, digital subtraction angiography, hemobilia, hepatic artery pseudoaneurysm, liver transplantation

## Abstract

**Background:**

Hepatic artery pseudoaneurysm (HAP) is a rare but life-threatening complication following liver transplantation (LT). The formation of an arterio-biliary fistula leading to hemobilia is exceptionally uncommon, particularly when manifesting several months post-transplantation. Diagnosis and therapeutic management remain highly challenging because achieving definitive hemostasis often conflicts with the critical need to preserve graft arterial perfusion.

**Case presentation:**

A 23-year-old male presented with recurrent hematemesis, hematochezia, and right upper quadrant pain 4 months after undergoing orthotopic LT for end-stage alcohol-related cirrhosis. While initial endoscopy pointed toward a duodenal bulb ulcer, a repeat endoscopic evaluation revealed fresh blood actively emerging from the major duodenal papilla, raising strong suspicion for hemobilia. Subsequent digital subtraction angiography (DSA) demonstrated a post-transplant HAP arising from the hilar hepatic arterial segment, communicating directly with the common bile duct, confirming an arterio-biliary fistula. Two sessions of transcatheter coil embolization achieved only temporary hemostasis. Following an early recurrence of bleeding, the patient underwent emergency surgery. The lesion was identified on the left hepatic artery near the porta hepatis. The surgical intervention included pseudoaneurysm resection, end-to-end hepatic artery reconstruction, and a Roux-en-Y biliary-enteric anastomosis. Despite an initially stable early postoperative course, the patient suffered a sudden circulatory collapse on postoperative day 6 and died despite aggressive resuscitation.

**Conclusions:**

Hemobilia should be considered in liver transplant recipients with recurrent or unexplained gastrointestinal bleeding, particularly when blood is observed emerging from the major duodenal papilla. DSA should be performed promptly when clinical suspicion remains high, even if post-embolization CTA is inconclusive. Recurrent bleeding after embolization may indicate persistent fistulous communication and should prompt early multidisciplinary reassessment of definitive treatment options.

## Background

Hepatic artery pseudoaneurysm (HAP) is an uncommon yet potentially catastrophic vascular complication following liver transplantation (LT), carrying a reported incidence of 0.3%–2.6% (1). Rupture of the pseudoaneurysm can precipitate intra-abdominal hemorrhage, hemobilia, graft ischemia, or sudden death. Proposed etiological factors include mechanical arterial wall injury, anastomotic technical complications, localized infection, biliary leakage, and immunosuppression (1, 2). While most post-transplant HAPs are detected within the first 2 months after surgery, delayed presentations are far less frequent (1, 3). Hemobilia represents an extremely rare clinical presentation of HAP. Its clinical manifestations are often insidious and nonspecific, making it prone to misdiagnosis as conventional upper gastrointestinal bleeding during the early stages (4). Computed tomography angiography (CTA) and digital subtraction angiography (DSA) serve as crucial diagnostic modalities for evaluating suspected HAP. Among these, DSA is highly valuable as it concurrently facilitates localization of the vascular lesion, identification of abnormal communications, and immediate endovascular intervention (2, 5). However, when HAP is further complicated by a direct arterio-biliary fistula, both diagnostic confirmation and therapeutic decision-making become significantly more complex.

Although post-transplant HAP has been documented previously, delayed hemobilia arising from a direct communication between an HAP and the biliary tree remains exceedingly rare. The diagnosis is frequently obscured or delayed by coexisting gastrointestinal mucosal lesions, and the optimal management strategy remains controversial when recurrent bleeding occurs despite repeated endovascular interventions. Here, we report a fatal case of delayed hemobilia secondary to an HAP with an arterio-biliary fistula presenting 4 months after orthotopic LT. This case highlights the critical diagnostic relevance of endoscopic papillary bleeding, the potential pitfalls of an apparently negative post-embolization CTA, and the clinical dilemma of balancing definitive hemostasis with the preservation of graft arterial perfusion.

## Case presentation

A 23-year-old man with end-stage alcohol-related cirrhosis underwent deceased-donor orthotopic LT in 2024. Prior to transplantation, his course was complicated by recurrent hepatic decompensation requiring multiple sessions of plasma exchange, alongside a history of esophagogastric variceal bleeding managed endoscopically in 2023. During transplantation, a donor right hepatic artery arising from the superior mesenteric artery was identified. Hepatic arterial reconstruction therefore involved two end-to-end anastomoses: the donor common hepatic artery was anastomosed to the recipient right hepatic artery using 6-0 Prolene, and the donor right hepatic artery was anastomosed to the recipient gastroduodenal artery using 7-0 Prolene. No additional arterial anatomical variation was identified in the recipient. The portal vein and common bile duct were reconstructed by standard end-to-end anastomoses, and a modified piggyback technique was used for caval reconstruction. Because the original operative record did not document a measurable distance between the pseudoaneurysm and the arterial anastomosis, the exact aneurysm-to-anastomosis distance could not be determined retrospectively. No definite anastomotic disruption or dehiscence was documented. Postoperatively, the initial immunosuppressive regimen consisted of tacrolimus, enteric-coated mycophenolate sodium, and corticosteroids, with subsequent dose adjustment according to clinical status and drug concentrations. Early postoperative anti-infective therapy included meropenem (1 g every 8 h), vancomycin (1 g every 12 h), and voriconazole (0.2 g once daily). No antiplatelet or systemic anticoagulant therapy was documented during the immediate postoperative period.

During the early postoperative period, the patient developed fever from approximately POD 6 to POD 8, with a maximum temperature of 38.9 °C. Blood culture yielded Enterococcus faecium, although no definite anatomical source of bacteremia was identified. SARS-CoV-2 nucleic acid testing was also positive, whereas an extended respiratory pathogen panel was negative. Inflammatory markers were elevated during the febrile episode, with C-reactive protein reaching 64.2 mg/L and procalcitonin 0.46 ng/mL. The patient received meropenem and vancomycin, together with antifungal and SARS-CoV-2–directed antiviral therapy. The fever resolved by POD 9. Abdominal ultrasonography and contrast-enhanced CT demonstrated postoperative intra-abdominal fluid collections, but no definite biliary anastomotic leakage or localized intra-abdominal infectious focus was identified. Bile culture was negative. The overall clinical course from liver transplantation to the final outcome is summarized in Table 1.

**Table 1:** Clinical timeline of the patient from liver transplantation to the final outcome.

Date/time	Clinical event
At transplantation	Deceased-donor orthotopic liver transplantation.
POD 6–8	Early postoperative fever (maximum 38.9 °C) with Enterococcus faecium bacteremia and positive SARS-CoV-2 nucleic acid testing. Broad-spectrum antimicrobial and antiviral therapy was administered, with resolution of fever by POD 9.
4 months after LT	Recurrent hematemesis and hematochezia; endoscopy suggested hemobilia.
First embolization	HAP identified on DSA; coil embolization performed. Hemoglobin 64 g/L.
6 days after first embolization	Recurrent bleeding; arterio-biliary fistula confirmed; second embolization performed.
Discharge	No active bleeding or fever; hemodynamically stable; hemoglobin 82 g/L.
Three days after discharge	Recurrent abdominal pain and hematochezia; emergency surgery was planned.
Emergency surgery	Pseudoaneurysm resection, hepatic artery reconstruction, and biliary-enteric reconstruction.
POD 6	Sudden circulatory collapse with severe anemia and metabolic acidosis; death despite resuscitation.

As part of routine early postoperative surveillance after LT, hepatic arterial patency was monitored by serial Doppler ultrasonography, with additional examinations performed as clinically indicated. In this patient, these examinations demonstrated patent hepatic arterial flow, although transient increases in the hepatic arterial resistance index and mild arterial wall or anastomotic flow abnormalities were observed. Subsequent Doppler follow-up showed improvement, with no focal hepatic arterial lesion or pseudoaneurysm identified during this period. Longitudinal laboratory findings and tacrolimus concentrations during the first four months after LT are summarized in Supplementary Table S1.

Approximately 4 months post-transplantation, the patient developed recurrent episodes of bright-red hematemesis (approximately 50 mL per episode) followed by repeated dark-red hematochezia. The bleeding episodes were accompanied by a persistent, dull right upper quadrant abdominal pain, acid regurgitation, fatigue, dizziness, blurred vision, and diaphoresis. Given his past medical history, a recurrent portal hypertensive variceal hemorrhage was initially suspected. An initial upper gastrointestinal endoscopy revealed a duodenal bulb ulcer in the active stage, which was initially interpreted as the likely source of bleeding. However, no active bleeding from the ulcer was observed, and no endoscopic hemostatic treatment was performed. Nevertheless, gastrointestinal bleeding and progressive anemia persisted despite intensive supportive care. A repeat endoscopy demonstrated a large, dark-red blood clot adherent to the major duodenal papilla. Following careful removal of the clot, fresh blood was observed actively oozing from the papillary orifice, strongly indicating an intrahepatic or biliary vascular source (Figure 1A).

**Figure 1:**
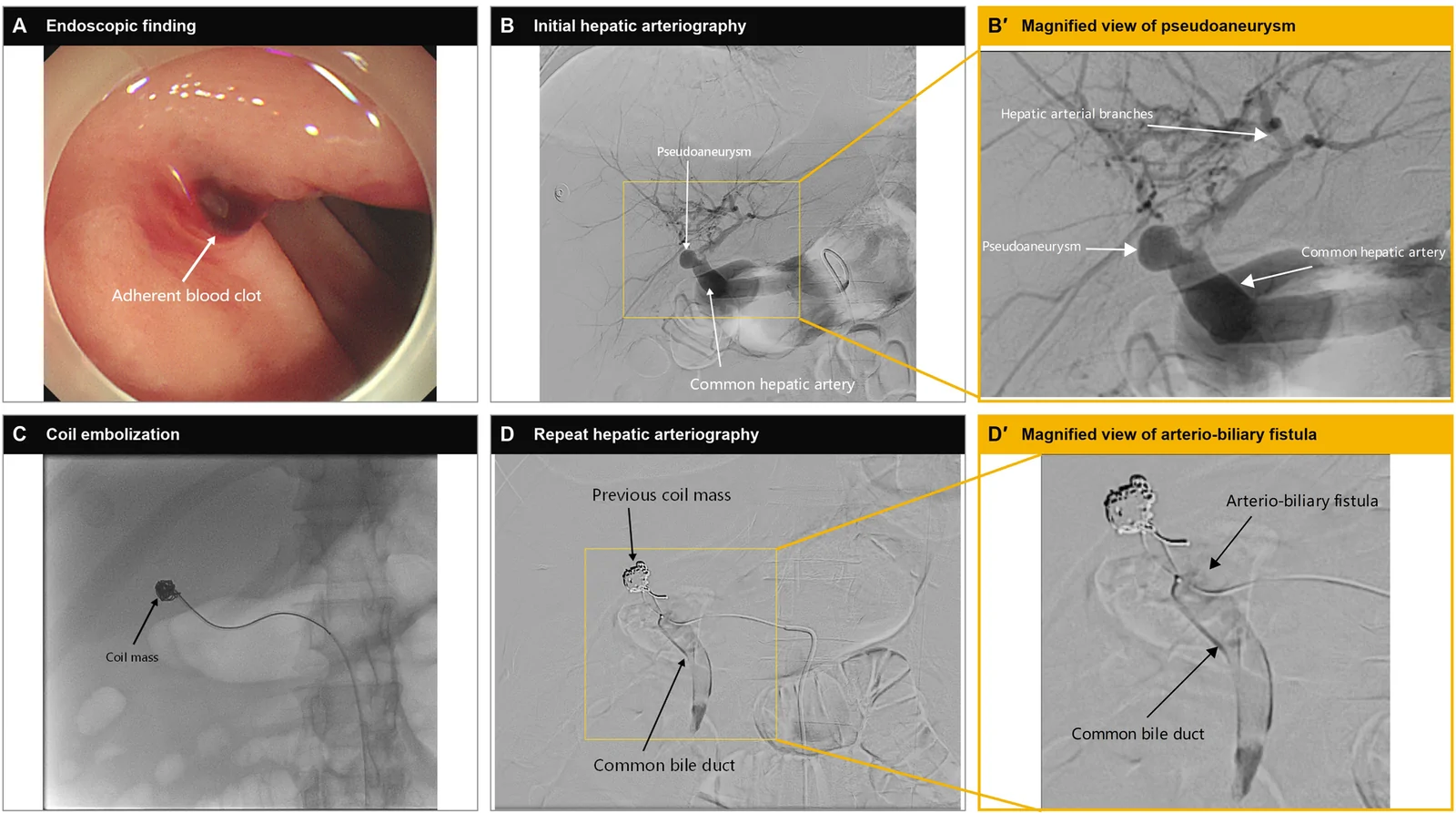
Endoscopic and arteriographic findings. **(A)** Upper gastrointestinal endoscopy showing a dark-red blood clot adherent to the major duodenal papilla, suggesting hemobilia. **(B)** Initial selective hepatic arteriography showing a pseudoaneurysm in the common hepatic artery region; the boxed area indicates the region enlarged in panel **B′**. **(B′)** Magnified view of the boxed area in panel **B**, demonstrating the pseudoaneurysm, common hepatic artery, and adjacent hepatic arterial branches. **(C)** Fluoroscopic image during the first endovascular procedure showing the coil mass within the treated pseudoaneurysm sac. **(D)** Repeat hepatic arteriography showing the previous coil mass and contrast opacification of the common bile duct; the boxed area indicates the region enlarged in panel **D′**. **(D′)** Magnified view demonstrating the fistulous communication between the hepatic artery and common bile duct, confirming an arterio-biliary fistula.

The patient subsequently underwent two urgent DSA and transcatheter intervention procedures 6 days apart. Initial angiography identified a pseudoaneurysm originating from the hilar segment of the hepatic artery (Figures 1B,B′). During the first procedure, the microcatheter was advanced superselectively into the pseudoaneurysm sac, which was embolized with detachable coils (Figure 1C). During the second angiographic session, contrast media was observed flowing directly from the pseudoaneurysm into the common bile duct, confirming the presence of an arterio-biliary fistula (Figures 1D,D′). The fistulous opening was subsequently superselectively catheterized and embolized with two detachable fibered coils; subsequent angiography showed no further opacification of the common bile duct, while hepatic arterial flow remained patent.

Although two sessions of endovascular coil embolization achieved temporary hemostasis, a multidisciplinary clinical review concluded that complete occlusion of the hepatic artery would likely trigger catastrophic graft ischemia and acute liver failure, while open surgical reconstruction carried a prohibitively high immediate perioperative risk. Consequently, further invasive interventions were deferred. A post-embolization three-dimensional CTA demonstrated a dense focus in the hepatic hilar region. Evaluation of the adjacent structures was partially limited by metal-related artifacts, and no definite residual filling of the pseudoaneurysm was identified (Figure 2).

**Figure 2:**
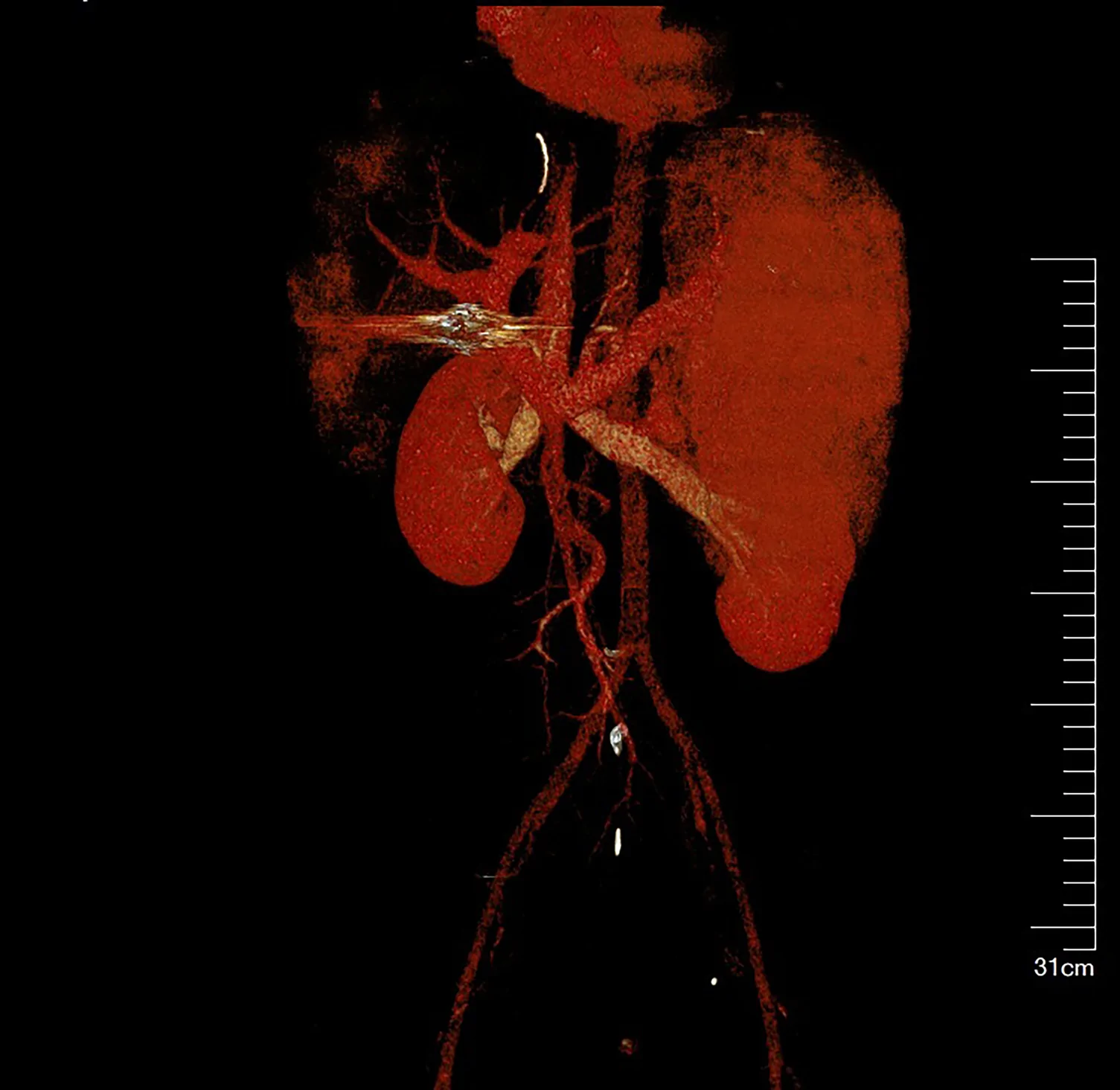
Post-embolization computed tomography angiography. Three-dimensional CTA reconstruction showing a dense focus in the hepatic hilar region following embolization. Evaluation of the adjacent hilar structures was partially limited by metal-related artifacts, with no definite residual filling of the pseudoaneurysm identified.

Following the second embolization, the patient remained hospitalized for management of persistent fever and suspected hepatobiliary infection. Before discharge, no further major overt bleeding had been documented for more than 10 days, and he was afebrile and hemodynamically stable. His hemoglobin was 82 g/L, with procalcitonin 0.39 ng/mL and C-reactive protein 29.8 mg/L. Multidisciplinary imaging review showed patent major graft vessels. He was therefore discharged on oral antibiotic therapy with scheduled gastroenterology and transplant follow-up.

Three days after discharge the patient was readmitted with recurrent abdominal pain and hematochezia. His temperature was 38.6 °C on admission. Ultrasonography performed one day earlier showed multiple hypoechoic lesions in the right hepatic lobe, considered possibly inflammatory, with mild intrahepatic bile duct dilatation, while hepatic arterial flow remained patent. Given the recurrence of bleeding after two previous endovascular embolization procedures and the known hepatic artery pseudoaneurysm with an arterio-biliary fistula, durable control by further conservative management was considered unlikely. Emergency surgical exploration was therefore undertaken.

Surgical exploration revealed dense, extensive adhesions throughout the upper abdomen. Following meticulous dissection of the porta hepatis, a pseudoaneurysm measuring approximately 1.5 cm in diameter was identified arising from the left hepatic artery, located roughly 2 cm from the porta hepatis, where it was directly compressing the common bile duct. Resection of the pseudoaneurysm exposed a clear fistulous opening communicating between the hepatic artery and the biliary tract. The resected specimen was submitted for histopathological examination; however, no final histopathological report was available, and no intraoperative microbiological cultures were documented from the pseudoaneurysm wall, thrombus, arterial tissue, bile, or surrounding tissue. The arterial ends were carefully trimmed and reconstructed via an end-to-end anastomosis using 6-0 Prolene sutures. Intraoperative Doppler ultrasonography confirmed satisfactory and patent hepatic arterial flow. The severely dilated common bile duct was resected; the distal ductal stump was closed, and a Roux-en-Y biliary-enteric reconstruction was performed using the proximal biliary remnant. The estimated intraoperative blood loss was approximately 800 mL. The patient was transfused with 6 units of packed red blood cells and 300 mL of pathogen-reduced fresh frozen plasma.

Postoperatively, the graft was monitored by serial Doppler ultrasonography. Hepatic arterial flow remained detectable throughout POD0–5, with no definite evidence of hepatic artery thrombosis. The hepatic arterial resistance index was 0.65 on POD0 and subsequently remained relatively low (approximately 0.42–0.44 on POD1–3), with transiently increased flow velocities. On POD5, the peak systolic velocity increased to approximately 227 cm/s with an RI of 0.60, while multiple hypoechoic areas in the right hepatic lobe were considered possibly ischemic. Biochemically, the patient demonstrated early postoperative hepatic injury, with AST and ALT peaking at 566 and 411.5 IU/L, respectively, and total bilirubin at 158.4 μmol/L on POD0. These abnormalities subsequently improved, with AST 58.6 IU/L, ALT 51.6 IU/L, and total bilirubin 53.1 μmol/L by POD5. INR improved from 1.86 on POD0 to approximately 1.12–1.20 during POD2–5, and serum creatinine decreased from a peak of 173.3 μmol/L on POD1 to 98.5 μmol/L on POD5. Severe anemia persisted, with hemoglobin ranging from approximately 50–75 g/L during POD1–5. The white blood cell count normalized after an early postoperative increase. Serial lactate and procalcitonin measurements were not available during this interval.

On postoperative day 6, the patient suddenly developed agitation followed by coma, hypotension, bradycardia, and severe hypoxemia. Emergency testing showed profound circulatory collapse, including pH 6.80, lactate 9.7 mmol/L, hemoglobin 30 g/L, and INR 2.92. Recurrent hemobilia with hemorrhagic shock was considered the most likely cause by the treating team and subsequent mortality review. However, because no emergency imaging, surgical re-exploration, or autopsy was performed, the exact cause of death remained unconfirmed, and intra-abdominal hemorrhage, hepatic artery thrombosis, severe infection/septic shock, or other acute postoperative complications could not be excluded.

## Discussion

This case illustrates a rare and clinically challenging form of delayed post-transplant hemobilia caused by a hepatic artery pseudoaneurysm with direct arterio-biliary communication. Its principal educational features include the delayed presentation 4 months after transplantation, the diagnostic distraction caused by a coexisting duodenal ulcer, direct endoscopic visualization of papillary bleeding, recurrent hemorrhage after two embolization procedures, discordance between post-embolization CTA and DSA findings, and the difficulty of achieving durable hemostasis while preserving graft arterial perfusion. Among previously reported cases with available demographic data, this patient was the youngest. The case is also distinctive because it provides a detailed account of combined hepatic arterial and biliary reconstruction and documents a fatal outcome despite apparently successful intraoperative restoration of hepatic arterial flow.

HAP after LT is uncommon but is associated with substantial risks of graft loss and mortality (6, 7). A 2025 systematic review of 11 adult case series involving 118 patients found hemorrhagic shock or luminal bleeding to be the most frequent presentation, with bile leakage and localized infection commonly reported (8). Earlier radiologic observations and published Chinese experience similarly documented variable presentations, including hemobilia and biliary complications, and highlighted the competing risks of arterial occlusion and surgical treatment (9–11). The wide variation in the interval from transplantation to diagnosis indicates that HAP should not be regarded exclusively as an early postoperative complication. In the present patient, symptoms developed approximately 4 months after transplantation.

Notably, the patient experienced an early postoperative febrile episode with Enterococcus faecium bacteremia. During the same period, serial Doppler ultrasonography demonstrated patent hepatic arterial flow but transiently increased hepatic arterial resistance indices and nonspecific arterial wall/anastomotic abnormalities, without evidence of a pseudoaneurysm. During the later clinical course, recurrent fever and imaging findings suggestive of inflammatory hepatic lesions raised further concern for a concomitant hepatobiliary infectious process. Therefore, an infectious contribution to the development or persistence of the pseudoaneurysm could not be excluded. However, because no final histopathological result was available and microbiological culture of the pseudoaneurysm wall was not performed, the lesion could not be definitively classified as an infectious (mycotic) pseudoaneurysm.

The pathogenesis of HAP in this patient was likely multifactorial. Post-transplant HAP may be associated with procedural or local arterial injury, while hepatobiliary infection may also contribute to arterial-wall disruption (1, 2). In this patient, the complex hepatic arterial reconstruction may have created a local substrate for vascular injury, and the early postoperative *Enterococcus faecium* bacteremia together with later recurrent fever and suspected hepatobiliary infection raises the possibility of an additional infection-related contribution. Infectious hepatic arterial aneurysms have been reported after liver transplantation (12); however, an infectious or mycotic pseudoaneurysm could not be confirmed because aneurysm-wall culture was not performed and no final histopathological result was available. No definite bile leakage was documented. Nevertheless, the operative finding that the pseudoaneurysm directly compressed the common bile duct with a visible arterio-biliary fistulous opening supports, although does not prove, a possible sequence in which progressive arterial-wall disruption and pseudoaneurysm enlargement subsequently eroded into the adjacent bile duct, resulting in the arterio-biliary fistula and recurrent hemobilia (4).

From a clinical perspective, infection- or biliary complication–associated HAP should be considered when liver transplant recipients develop unexplained fever, bacteremia, biliary leakage or perihepatic collections, particularly when these findings are accompanied by gastrointestinal bleeding or hemobilia (8, 12). Doppler ultrasonography is useful for surveillance of hepatic arterial patency, whereas CTA and, when clinical suspicion remains high, DSA are important for defining the vascular lesion and any associated arterio-biliary communication (2). Once established, HAP may enlarge, rupture, or erode into adjacent biliary structures (1, 4). Management therefore requires not only hemorrhage control but also assessment and treatment of any associated infection or biliary source, with endovascular or surgical treatment selected according to vascular anatomy, graft perfusion, and clinical stability (8).

Published case reports and small series describing HAP with documented arterio-biliary fistula after LT are summarized in Table 2. Most patients presented with hemobilia or other forms of gastrointestinal bleeding, commonly within the first 3 months after transplantation, although later presentations have also been reported. Reported treatment strategies included embolization, covered-stent placement, hepatic artery ligation, arterial reconstruction, graft hepatectomy, and retransplantation.

**Table 2:** Clinical characteristics of reported cases of hepatic artery pseudoaneurysm with arterio-biliary fistula after liver transplantation, including the present case.

Study	Age/sex	Interval after LT	Presentation	Treatment	Outcome
Houssin et al., 1988 (12)	NR	NR	NR	NR	NR
Langnas et al., 1991 (13)	NR	NR	NR	HA ligation or embolization	NR
Riedmann et al., 1995 (14)	54/M	2 months	Melena, jaundice, and hemorrhagic shock	HAP resection, arterial repair, and biliary reconstruction	Alive at 2 years
Leonardi et al., 2001 (15)	NR	POD 10	Hemobilia	NR	NR
Almogy et al., 2002 (16)	45/M	31 days	Melena, severe weakness, hemorrhagic shock, and hemobilia	Surgical ligation and biliary reconstruction followed by coil embolization	Alive and well at 10 months
Álamo et al., 2005 (17)	43/M	95 days	Massive gastrointestinal bleeding and shock	Emergency graft hepatectomy	Died
Finley et al., 2005 (18)	NR	NR	Hemobilia	NR	NR
Thorat et al., 2017, case 1 (5)	51/M	4 months	Recurrent gastrointestinal bleeding and hemobilia	Coil embolization	Recovered
Thorat et al., 2017, case 2 (5)	52/NR	10 weeks	Fever, liver abscess, melena, and hemobilia	Angioplasty and covered-stent placement	Recovered
Harrison et al., 2017, patient 2 (19)	55/M	72 days	NR	NR	Survived
Harrison et al., 2017, patient 4 (19)	58/M	28 days	NR	NR	Survived
Harrison et al., 2017, patient 7 (19)	62/F	41 days	NR	NR	Survived
Páez-Carpio et al., 2020 (20)	56/M	2 months	Hematemesis, melena, and papillary bleeding	Covered coronary stent	Graft preserved at 18 months
Shen et al., 2024 (21)	66/M	POD 20	Massive hemobilia during ERCP	Balloon tamponade, biliary stent, and surgery	Survived
Pinlac et al., 2024 (22)	40/M	NR	Hemobilia	Covered-stent placement	Patent HA at 6 months
Present case	23/M	4 months	Hematemesis, hematochezia, and papillary bleeding	Two coil embolizations followed by arterial and biliary reconstruction	Died on POD 6

ERCP, endoscopic retrograde cholangiopancreatography; F, female; HA, hepatic artery; HAP, hepatic artery pseudoaneurysm; LT, liver transplantation; M, male; NR, not reported; POD, postoperative day.

Among cases with available demographic data, the present patient was the youngest. Although the 4-month interval represented a relatively delayed presentation, Thorat et al. reported a case at a similar interval (5). Therefore, the main contribution of the present report lies not in timing alone, but in the combination of a misleading duodenal ulcer, direct endoscopic visualization of papillary bleeding, recurrent hemorrhage after two embolization procedures, combined hepatic arterial and biliary reconstruction, and a fatal postoperative outcome. The marked heterogeneity of previously reported management strategies reflects differences in vascular anatomy, fistula location, graft perfusion, infection status, and local expertise (12–22).

The classic Quincke triad of abdominal pain, jaundice, and gastrointestinal bleeding occurs in only a minority of patients with hemobilia (23–25). Consequently, diagnostic delay is common when other plausible bleeding sources coexist. In this case, the history of variceal hemorrhage and the duodenal bulb ulcer provided convincing but misleading explanations for the bleeding. The decisive finding was fresh blood emerging from the major duodenal papilla on repeat endoscopy. Persistent bleeding despite treatment of an initially identified lesion should therefore prompt repeat endoscopic assessment, and papillary bleeding should trigger urgent evaluation for a hepatobiliary vascular source.

CTA and DSA play complementary roles in the evaluation of suspected HAP. CTA provides rapid, noninvasive assessment of the graft vasculature and surrounding anatomy, whereas DSA offers dynamic visualization of the feeding artery, contrast extravasation, and fistulous communication while permitting immediate endovascular treatment (2, 5, 26). In this patient, two DSA examinations demonstrated the pseudoaneurysm and its communication with the bile duct, whereas post-embolization three-dimensional CTA showed no definite residual lesion. Reduced contrast filling within the excluded sac and coil-related metal artifacts likely limited CTA assessment. Therefore, an inconclusive post-embolization CTA should not exclude persistent or recurrent HAP when bleeding continues or recurs; repeat DSA should be considered in this setting.

The management of HAP requires durable hemorrhage control without compromising graft arterial inflow. Transcatheter embolization is commonly used as first-line treatment, particularly in acute bleeding, but complete occlusion of the transplant hepatic artery may cause graft ischemia, biliary necrosis, and acute liver failure (5, 8, 22). Flow-preserving alternatives include covered-stent placement, open resection with arterial reconstruction, and urgent retransplantation in selected patients (21, 27, 28). Retransplantation was not considered in the present case because the dominant clinical problem was a localized vascular-biliary complication rather than graft failure, and liver function was not considered sufficiently impaired to warrant retransplantation. Given the very high operative risk, the multidisciplinary strategy remained focused on control of the arterio-biliary lesion while preserving the existing graft.

The feasibility of a covered stent depends on an adequate landing zone, vessel caliber and tortuosity, proximity to major bifurcations, and control of local infection or biliary contamination. In the present case, covered-stent placement was not documented as having been attempted, and the available records lacked detailed measurements of the vessel caliber and proximal and distal landing zones required to retrospectively determine its technical feasibility. Importantly, the interventional team documented that any further endovascular treatment would require complete hepatic arterial embolization with exclusion of the transplant arterial anastomosis, with a substantial risk of graft ischemia and liver failure.

The reason for recurrent bleeding after embolization could not be definitively established. Post-embolization CTA showed no definite residual filling of the pseudoaneurysm, and the available records did not document collateral arterial supply or definite recanalization. However, the arterio-biliary fistula had been directly demonstrated during the second angiographic procedure and was subsequently confirmed at surgery, suggesting that the pathological arterial-biliary communication had not been durably eliminated. Moreover, complete hepatic arterial occlusion was avoided because of the substantial risk of graft ischemia and liver failure (5, 8, 22). Therefore, persistent or intermittent perfusion of the pseudoaneurysm or fistulous tract may explain the temporary hemostasis followed by recurrent bleeding, although the precise mechanism remains uncertain. Emergency surgery enabled complete pseudoaneurysm resection, end-to-end reconstruction of the hepatic artery, and Roux-en-Y biliary-enteric reconstruction. Intraoperative Doppler ultrasonography demonstrated satisfactory hepatic arterial flow, suggesting technically successful restoration of graft arterial perfusion.

Hepatic artery thrombosis is an important vascular complication after hepatic arterial reconstruction and may lead to graft ischemia, biliary ischemia, graft dysfunction, and graft loss (29). Clinical presentation may be abrupt or initially nonspecific; therefore, serial Doppler ultrasonography and liver biochemical monitoring are important during the early postoperative period. When clinical deterioration occurs despite previously patent arterial flow, urgent vascular imaging should be considered. Early recognition is critical because treatment may require urgent revascularization or surgical revision, while retransplantation may be necessary when arterial flow cannot be restored or irreversible graft failure develops (29). Although the operative record did not document formal consideration of left hepatic artery ligation, preservation of hepatic arterial inflow was particularly relevant because the right hepatic artery could not be adequately explored intraoperatively. As direct end-to-end reconstruction was technically achievable after pseudoaneurysm resection, arterial continuity was restored rather than sacrificing the involved artery. Nevertheless, the patient developed fatal circulatory collapse on postoperative day 6. This discrepancy between apparently successful intraoperative reconstruction and the subsequent catastrophic outcome represents an important feature of the present case.

Severe metabolic acidosis, hyperlactatemia, profound anemia, coagulopathy, and acute kidney injury were consistent with catastrophic circulatory shock. Recurrent hemorrhage was clinically suspected but could not be confirmed. Acute thrombosis of the reconstructed hepatic artery with subsequent hepatic ischemia and graft failure was also an important differential diagnosis. Although serial Doppler examinations demonstrated detectable hepatic arterial flow through POD5 and liver biochemical indices had generally improved, acute hepatic artery thrombosis occurring at the time of deterioration on POD6 could not be excluded because no emergency vascular imaging, surgical re-exploration, or autopsy was performed. Severe infection/septic shock and other acute postoperative complications also remained possible.

### Limitations

This report has several limitations. The exact cause of the pseudoaneurysm could not be established. Although the resected specimen was submitted for histopathological examination, no final histopathological result was available, and microbiological culture of the pseudoaneurysm wall was not performed, precluding definitive confirmation or exclusion of an infectious etiology. Detailed procedural measurements and complete technical records needed to assess the feasibility of covered stent placement were unavailable. The source of the terminal circulatory collapse was not confirmed by emergency imaging, re-exploration, or autopsy. Finally, conclusions regarding the optimal timing of surgery or the comparative effectiveness of treatment strategies cannot be drawn from a single case.

## Conclusions

This case highlights that an HAP complicated by an arterio-biliary fistula represents a rare but potentially catastrophic cause of delayed hemobilia after liver transplantation. In liver transplant recipients presenting with recurrent, unexplained gastrointestinal hemorrhage—especially when fresh blood is endoscopically visualized oozing from the major duodenal papilla—hemobilia must be actively suspected, and urgent angiographic evaluation is warranted. Negative or ambiguous post-embolization CTA findings should be interpreted with extreme caution if clinical suspicion remains high. Therapeutic management requires customized, multidisciplinary decision-making aimed at achieving definitive hemostasis while preserving graft arterial perfusion. Further accumulation of multicenter clinical data is necessary to clarify the optimal diagnostic workflows and therapeutic algorithms for this uncommon post-transplant complication.

## Data Availability

The original contributions presented in the study are included in the article/Supplementary Material, further inquiries can be directed to the corresponding authors.
