# Supplementary material for: Case Report: Delayed hemobilia caused by hepatic artery pseudoaneurysm with arterio-biliary fistula after liver transplantation
Source: Front Med (Lausanne). 2026 Oct 13;13:1940000. doi: 10.3389/fmed.2026.1940000 (PMC13709982; doi:10.3389/fmed.2026.1940000)
Supplement: Supplementary file 1 [file Datasheet1.pdf]

CARE Checklist - 2016: Information for writing a case report

Topic	Item	Checklist item description	Location in latest manuscript
**Title**	**1**	The words "case report" should be in the title along with the area of focus	Title
**Key Words**	**2**	Four to seven key words - include "case report" as one of the key words	Keywords
**Abstract**	**3a**	Background: What does this case report add to the medical literature?	Abstract - Background
	**3b**	Case summary: chief complaint, diagnoses, interventions, and outcomes	Abstract - Case presentation
	**3c**	Conclusion: What is the main "take-away" lesson from this case?	Abstract - Conclusions
**Introduction**	**4**	The current standard of care and contributions of this case - with references (1-2 paragraphs)	Background
**Timeline**	**5**	Information from this case report organized into a timeline (table or figure)	Case presentation (Table 1)
**Patient Information**	**6a**	De-identified demographic and other patient or client specific information	Case presentation
	**6b**	Chief complaint - what prompted this visit?	Case presentation
	**6c**	Relevant history including past interventions and outcomes	Case presentation
**Physical Exam**	**7**	Relevant physical examination findings	Not explicitly reported
**Diagnostic Assessment**	**8a**	Evaluations such as surveys, laboratory testing, imaging, etc.	Case presentation
	**8b**	Diagnostic reasoning including other diagnoses considered and challenges	Case presentation
	**8c**	Consider tables or figures linking assessment, diagnoses and interventions	Figure legends (Figures 1-2)
	**8d**	Prognostic characteristics where applicable	Not explicitly reported
**Interventions**	**9a**	Types such as life-style recommendations, treatments, medications, surgery	Case presentation
	**9b**	Intervention administration such as dosage, frequency and duration	Case presentation
	**9c**	Note changes in intervention with explanation	Case presentation
	**9d**	Other concurrent interventions	Case presentation
**Follow-up and Outcomes**	**10a**	Clinician assessment (and patient or client assessed outcomes when appropriate)	Case presentation
	**10b**	Important follow-up diagnostic evaluations	Case presentation
	**10c**	Assessment of intervention adherence and tolerability, including adverse events	Adverse outcome: Case presentation; adherence/tolerability not reported
**Discussion**	**11a**	Strengths and limitations in your approach to this case	Discussion; Limitations
	**11b**	Specify how this case report informs practice or Clinical Practice Guidelines (CPG)	Discussion; Conclusions
	**11c**	How does this case report suggest a testable hypothesis?	Not explicitly stated
	**11d**	Conclusions and rationale	Conclusions
**Patient Perspective**	**12**	When appropriate include the assessment of the patient or client on this episode of care	Not reported
**Informed Consent**	**13**	Informed consent from the person who is the subject of this case report is required by most journals	Ethics statement
**Additional Information**	**14**	Acknowledgement section; Competing Interests; IRB approval when required	Ethics statement; Funding; Conflict of interest

Completed using section names in the latest manuscript. Items marked 'not reported' or 'not explicitly stated' were not inferred beyond the submitted text.
